# Apigenin reduces the suppressive effect of exosomes derived from irritable bowel syndrome patients on the autophagy of human colon epithelial cells by promoting ATG14

**DOI:** 10.1186/s12957-023-02963-5

**Published:** 2023-03-14

**Authors:** Rui Fu, Saiyue Liu, Mingjin Zhu, Jiajie Zhu, Mingxian Chen

**Affiliations:** 1 Department of Gastroenterology, Tongde Hospital of Zhejiang Province, Gucui Road 234, Xihu District, Hangzhou, Zhejiang 310012 People’s Republic of China; 2 Department of Adverse Drug Reaction Monitoring, Zhejiang Province Center of Adverse Drug Reaction Monitoring, Hangzhou, Zhejiang 310012 People’s Republic of China

**Keywords:** Apigenin, Exosome, Irritable bowel syndrome, Autophagy, MicroRNA

## Abstract

**Background:**

Inflammatory bowel disease (IBS) is a chronic disorder of the gastrointestinal tract. Exosomes have been involved in various pathological processes including IBS. Apigenin has been reported to suppress inflammatory bowel disease (IBS). However, the regulatory roles of exosomes derived from IBS patients (IBS-exos) on human colon epithelial cells are still unclear.

**Methods:**

Exosomes were collected from IBS patients (IBS-exos) and co-cultured with CACO-2 cells. Apigenin was used to treat IBS-exos-treated CACO-2 cells. By exploring the public data bank, we figured out the regulators control the autophagy of CACO-2 cells.

**Results:**

Administration of apigenin dose-dependently abolished the inhibitory effect of IBS-exo on the autophagy of CACO-2 cells. A mechanistic study showed that miR-148b-3p bound to 3′UTR to suppress ATG14 and decrease autophagy. Moreover, results suggested that ATG14 overexpression promoted the autophagy of CACO-2 cells in the presence of miR-148b-3p mimic.

**Conclusion:**

The current study showed that apigenin dose-dependently abolished the inhibitory effect of IBS-exo on CACO-2 cell autophagy by regulating miR-148b-3p/ATG14 signaling.

## Introduction

Irritable bowel syndrome (IBS) is characterized by abdominal pain and alterations in bowel habits [1]. The prevalence rates of IBS in the USA and Canada are reported as about 12% [2]. Studies have indicated that IBS patients have a poor life quality and heavily use the health care system [3, 4].

Apigenin, one of the most studied phenolics, is present principally as glycosylated in significant amount in vegetables, fruits, and herbs [5]. Apigenin has been used as an antioxidant and anti-inflammatory [6, 7]. Apigenin was also reported to suppress various human cancers [8]. A recent study also demonstrated that apigenin is involved in anti-inflammation and autophagy and suppresses IBS [9, 10]. However, the role of apigenin in the autophagy of human colon epithelial cells is unclear.

Exosomes, 30–120-nm membrane-derived vesicles containing DNAs, mRNAs, microRNAs, or proteins, participate in cell communication and protein/RNA delivery [11, 12]. Exosomes have been shown to be secreted by a broad spectrum of cells and regulate the pathological development of numerous diseases [13, 14]. For example, exosomes secreted by mesenchymal stem cell (MSC) reduce myocardial ischemia/reperfusion injury [15]. Gallet et al. have shown that cardiosphere-derived cell-secreted exosomes reduce scarring and alleviate myocardial infarction [16]. Irritable bowel syndrome (IBS) is a chronic disorder of the intestines [1]. However, the regulatory relationship between exosomes derived from IBS patients (IBS-exos) and human colon epithelial cells is still unclear.

Autophagy is a cell survival mechanism which adapts cells to metabolic stresses [17]. Autophagy plays an important role in different cellular processes [18, 19]. Studies also demonstrated that autophagy plays a protective role against some human diseases, and autophagy dysfunction has previously been associated with a variety of diseases including cancer, neurodegeneration, and IBS [18, 20–22]. Studies also indicated that intestinal epithelial cells constitute the first physical barrier to protect the intestinal mucosa from injury, and the activation of intestinal epithelial cell autophagy is essential to maintain intestine function [23, 24]. However, the relationship between IBS-exos and the autophagy of human colon epithelial cells remains to be elucidated.

miR-148b-3p involves in different biological processes including autophagy and apoptosis. For instance, overexpressing miR-148b-3p down-regulated the viability, but increased the apoptosis of hypoxia/reoxygenation-treated cardiomyocytes [25]. A study also indicated that miR-148b-3p regulated pancreatic autophagy via suppression of autophagy elated 12 (ATG12) [26]. miR-148a has been shown to regulate autophagy by down-regulating IL-6/STAT3 signaling [27]. However, the function of miR-148b-3p in the autophagy of human colon epithelial cells is largely unknown.

Autophagy-related 14 (ATG14) play a very important role in autophagy by directing Complex I to function in autophagy by regulating its localization [28]. Xiong et al. have shown that ATG14 plays a critical role in hepatic autophagy and lipid metabolism [29]. Diao et al. indicated that ATG14 enhances membrane tethering and fusion of autophagosomes to endolysosomes [30]. But the role of ATG14 in the autophagy of human colon epithelial cells is rarely studied.

This study aims to explore the relationship between IBS-exos and autophagy of human colon epithelial cells, and the effect of apigenin on autophagy in human colon epithelial cells, therefore providing data for a better understanding of the role of apigenin in autophagy and IBS.

## Materials and methods

### Human blood

The study was approved by the Ethics Committee of Tongde Hospital of Zhejiang Province. Fifteen blood samples of IBS patients diagnosed according to Rome III criteria or controls were used to isolate exosomes. Written informed consent was received.

### Cell culture

CACO-2 cells were purchased from Shanghai Biology Institute and maintained in DMEM (Gibco, Carlsbad, CA, USA) with 10% FBS (Gibco) in an incubator at 37°C plus 5% CO_2_ atmosphere.

### Isolation and characterization of exosomes

Exosome in serum was collected as described previously [31]. Briefly, the serums were initially centrifuged at 3000 g for 15 min, to remove cells and other debris, and then the supernatants were span at 10000 g for 20 min to remove shedding vesicles and other vesicles that were larger than exosomes. Finally, the supernatants were span at 100,000 g for 1h at 4°C. Pellets were re-suspended in PBS and characterized by transmission electron microscopy (TEM) and immuno-staining.

### Exosome uptake analysis

Exosomes were stained by green fluorescent linker PKH67 (UR52303, Umibio, Shanghai, China). One milliliter of exosomes (1 μg/mL) was incubated with 2 μL PKH67 for 25 min at room temperature. In order to bind excess dye, 2 mL of 0.5% BSA/PBS was added. The labeled exosomes were washed at 100,000 g for 1 h, and the exosome pellet was suspended with PBS and used for uptake experiments. CACO-2 cells were seeded (50,000/well) and treated by medium with/without PKH67-labeled exosomes for 24 h. DAPI was used to stain the nucleus. Uptaking was observed under a fluorescence microscope (Leica Microsystems, Wetzlar, Germany).

### qRT-PCR

RNA was isolated and reverse transcribed into cDNA (Invitrogen, Waltham, MA, USA). Q-PCR was done using the SYBR Green qPCR Master Mixes (Thermo Fisher, Rockford, IL, USA) as follows: 95°C for 10 min followed by 40 cycles of 95°C for 15 s and 60°C for 45 s. U6 or β-actin was used as control. The gene relative expression was calculated by the 2^−ΔΔCt^ formula. The primers were as follows (5′-3′):

- has-miR-148b-3p, F: CGCGTCAGTGCATCACAGAA, R: AGTGCAGGGTCCGAGGTATT;
- U6, F: CTCGCTTCGGCAGCACA, R: AACGCTTCACGAATTTGCGT.
- ATG14, F: TCATTATGAGCGTCTGGC, R: ATGCTGGTGTCTCCGTTG;
- β-actin, F: AATGCCTTCACGATGTTC, R: AGCCTGCTGTAATATTGC.

### Immunoblotting

Protein was isolated using RIPA lysis buffer (JRDUN, Shanghai, P.R. China), concentration-measured by an enhanced BCA protein assay kit (Thermo Fisher Scientific), separated by 10% SDS-PAGE, and immunoblotted to PVDF membranes (Millipore, Billerica, MA, USA), blocked with 5% nonfat dry milk for 1 h at room temperature, and probed with primary antibodies at 4°C overnight. After washing with PBST, the bolts were incubated with a second antibody for 1 h at 37°C. An enhanced chemiluminescence system (Tanon, Shanghai, P.R. China) was used to visualize protein. Primary antibodies’ information was provided as follows: CD9 (Ab92726, Abcam, St. Louis, MO, USA), CD81 (Ab109201, Abcam), ATG14 (Ab227849, Abcam), and GAPDH (60004-1-1G, Proteintech, UK).

### Overexpression of ATG14

The pLVX-puro containing ATG14 (ovTAG14) or vector (ovNC) alone were purchased from Genechem company (Shanghai, China). CACO-2 cells were transfected with the plasmids using Lipo2000, and the cells were analyzed 48 h after transfection.

### Immunofluorescent staining

To evaluate the expression of autophagy markers LC3, cells were fixed, blocked, and probed with anti-LC3 antibody overnight at 4°C. Cells were washed and incubated in fluorochrome-conjugated secondary antibody for 1 h in the dark. Nuclei were counterstained by 4′,6-diamidino-2-phenylindole, dihydrochloride (DAPI), and cells were observed under a fluorescent microscope.

### Dual-luciferase reporter gene assay

Binding sites of miR-148b-3p and ATG14 were predicted by TargetScan. According to the prediction, wild type and mut sequences were synthesized respectively and cloned to luciferase reporter vectors (pGL3-Basic). Then, WT 3′ UTR or Mut 3′UTR plasmid was co-transfected with miR-148b-3p inhibitor or mimics into CACO-2 cells. After 48 h of transfection, a dual-luciferase reporter gene kit (Beijing Yuanpinghao Biotechnology Co., Ltd.) was used to determine the luciferase activity of cells in each group.

### Statistical analysis

Prism7.0 (La Jolla, CA) was used to analyze the data. Data was expressed as mean ± SD. Comparisons were performed by *T*-test or one-way ANOVA with Tukey’s post hoc test. *P* values less than 0.05 were considered as significant.

## Results

### Isolation and identification of exosomes

**Fig. 1:**
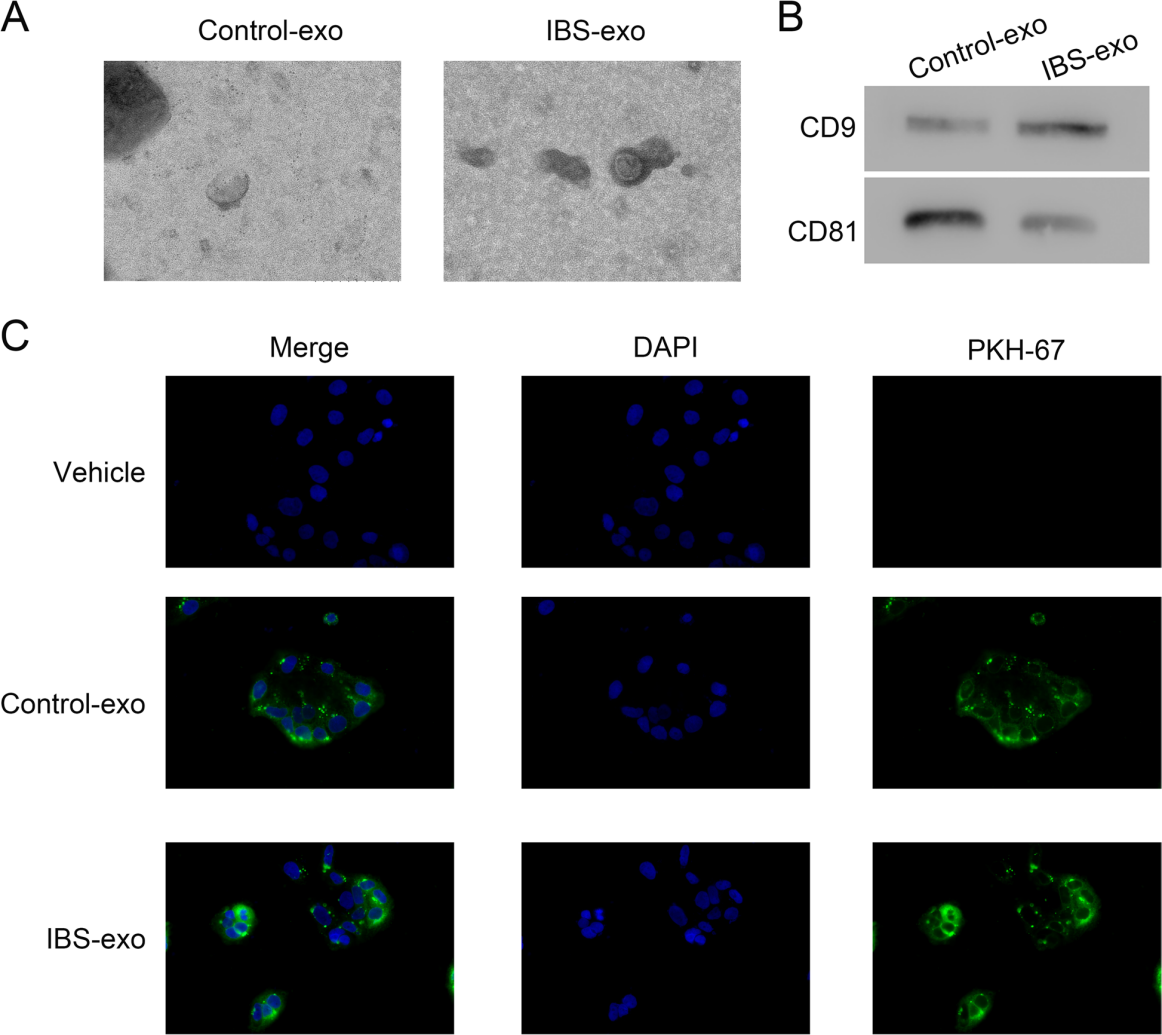
Isolation and characterization of exosomes. **A** Exosome morphology. **B** Western blotting detection of exosome markers, CD9 and CD81. ***p < 0.001 vs vehicle; !!!*p* < 0.001 vs oeNC + IBS-exo. **C** Uptake of exosomes by human colon epithelial Caco-2 cells was determined using PKH-67 dye

We collected serum samples from IBS patients and controls to extract exosomes. Exosomes from IBS patients (IBS-exo) or controls (control-exo) are shown in Fig. 1A. Immunoblotting further confirmed the expression of markers CD9 and CD81 (Fig. 1B). Co-culture assay showed that exosomes were up-taken by CACO-2 cells (Fig. 1C). This laid the foundation of this study.

### Exosome from IBS patients decreased the autophagy in CACO-2 cells

**Fig. 2:**
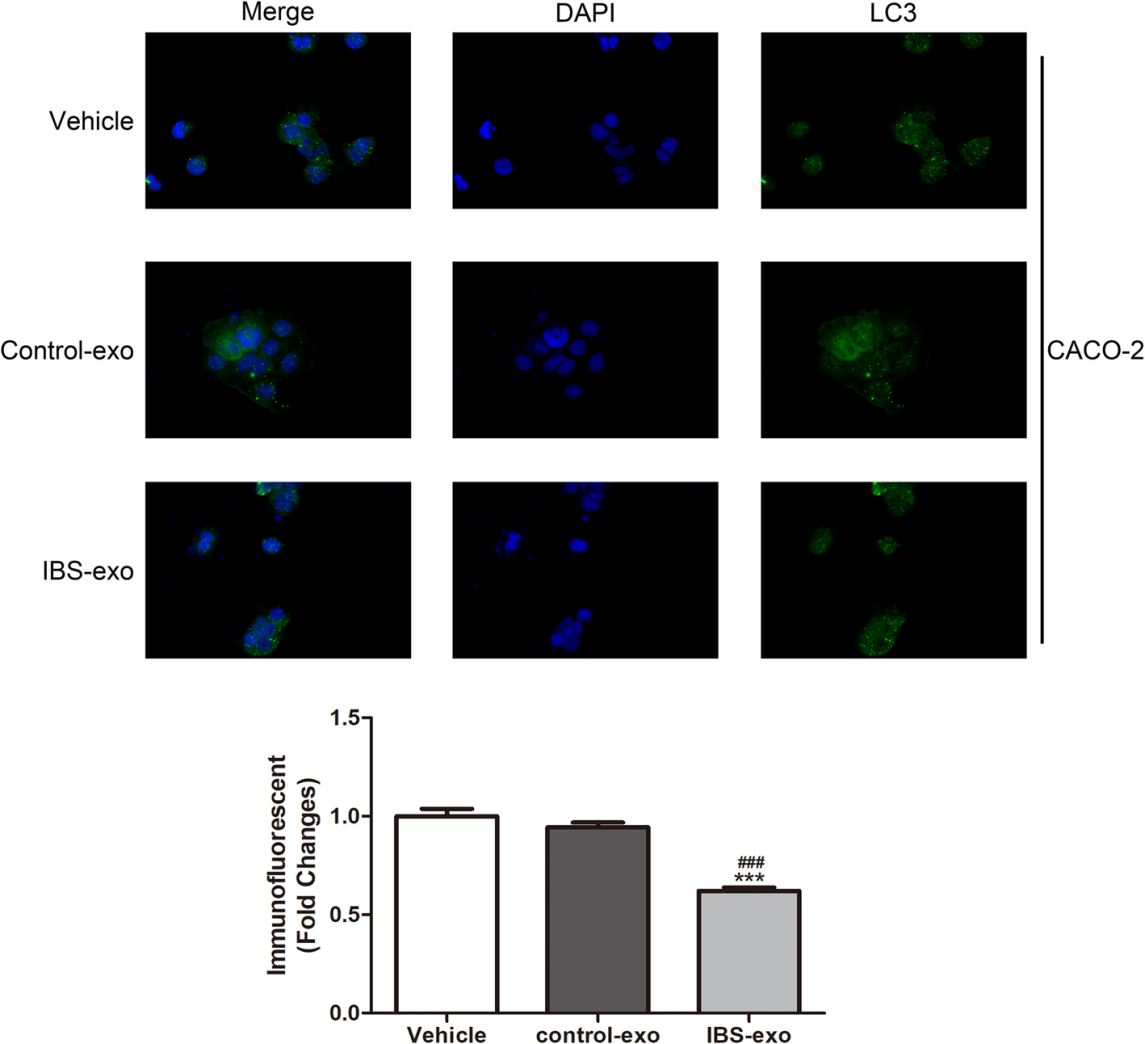
The autophagy of CACO-2 cells was decreased after co-cultured with IBS-exo. IMF staining examination of the effect of IBS-exo on CACO-2 autophagy

We next examined the effect of exosomes on autophagy of CACO-2, using LC3 as a maker of autophagy. Data revealed that co-culture with IBS-exos reduced autophagy in CACO-2 cells, compared to controls (Fig. 2).

### Apigenin dose-dependently abolished the inhibitory effect of IBS-exo on CACO-2 autophagy

**Fig. 3:**
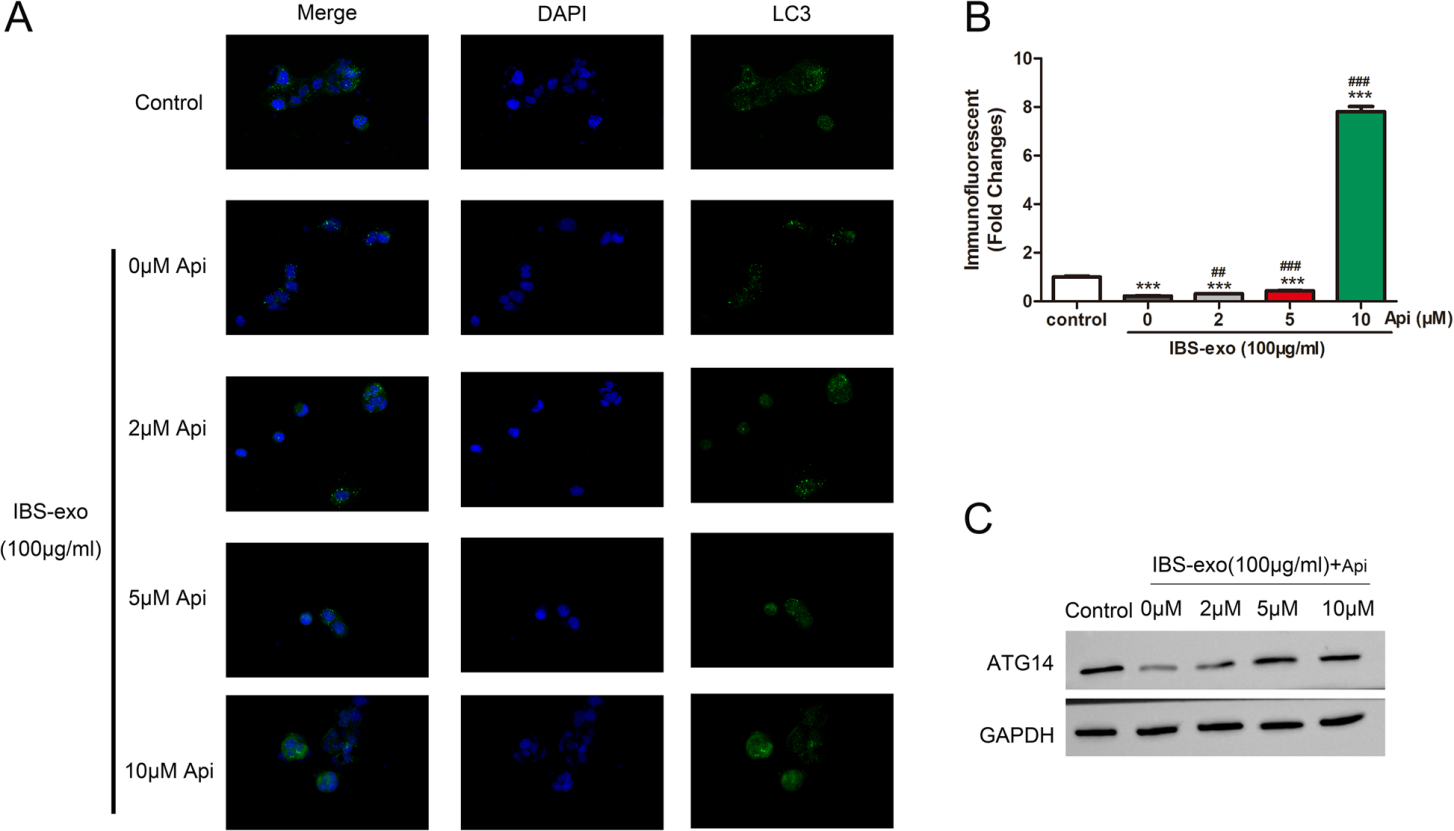
Apigenin dose-dependently promoted autophagy of CACO-2 cells that were co-cultured with IBS-exos. **A**, **B** IMF staining of LC3 was used to examine the autophagy of CACO-2 cells. ***p <0.01 vs control; ###*p* <0.001 vs IBS-exo. **C** ATG14 expression levels

In order to know whether apigenin affects CACO-2 autophagy, IMF staining was performed. Results suggested that apigenin dose-dependently abolished the inhibitory effect of IBS-exo CACO-2 autophagy (Fig. 3A, B). Western blotting showed that apigenin dose-dependently diminished IBS-exo-caused decrease of ATG14 protein in CACO-2 cells (Fig. 3C). This result indicates that apigenin abolished the suppression of autophagy by IBS-exos through regulating ATG14.

### miR-148b-3p bound 3′UTR of ATG14 to suppress its expression

**Fig. 4:**
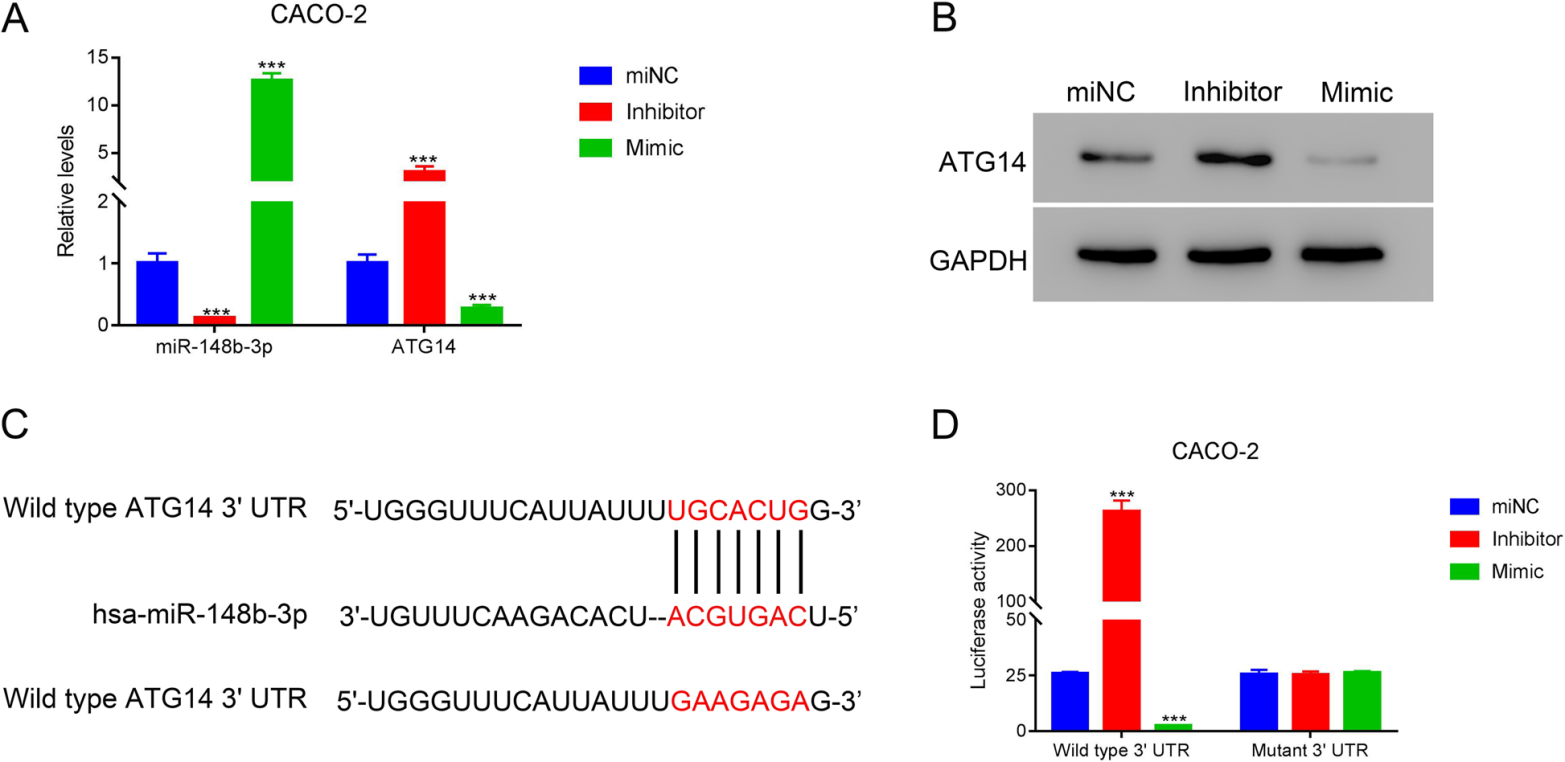
miR-148b-3p suppressed ATG14 via binding to 3′UTR. **A** Levels of miR-148b-3p and ATG14. **B** Protein levels of ATG14. **C** has-miR-148b-3p binding sites and corresponding mutation. **D** Dual-luciferase reporter gene verification of the binding of has-miR-148b-3p and ATG14. ***p <0.001 vs miNC

We further investigated the potential mechanisms by which apigenin promote autophagy of CACO-2 cells. By searching available data bank, we speculated ATG14 was a target of miR-148b-3p. So, we transfected CACO-2 cells with miR-148b-3p miNC, inhibitor, or mimic. QRT-PCR results indicated that transfection of miR-148b-3p inhibitor increased ATG14, while miR-148b-3p mimic decreased ATG14 (Fig. 4A). Transfection of miR-148b-3p mimic also decreased ATG14, while transfection of miR-148b-3p inhibitor increased ATG14 at protein level (Fig. 4B). Dual-luciferase reporter assay also confirmed the binding of has-miR-148b-3p and ATG14. Together, these findings indicated that miR-148b-3p suppressed ATG14 transcription through the binding on its 3′UTR.

### Overexpressing ATG14 promoted CACO-2 autophagy in the presence of miR-148b-3p mimic

**Fig. 5:**
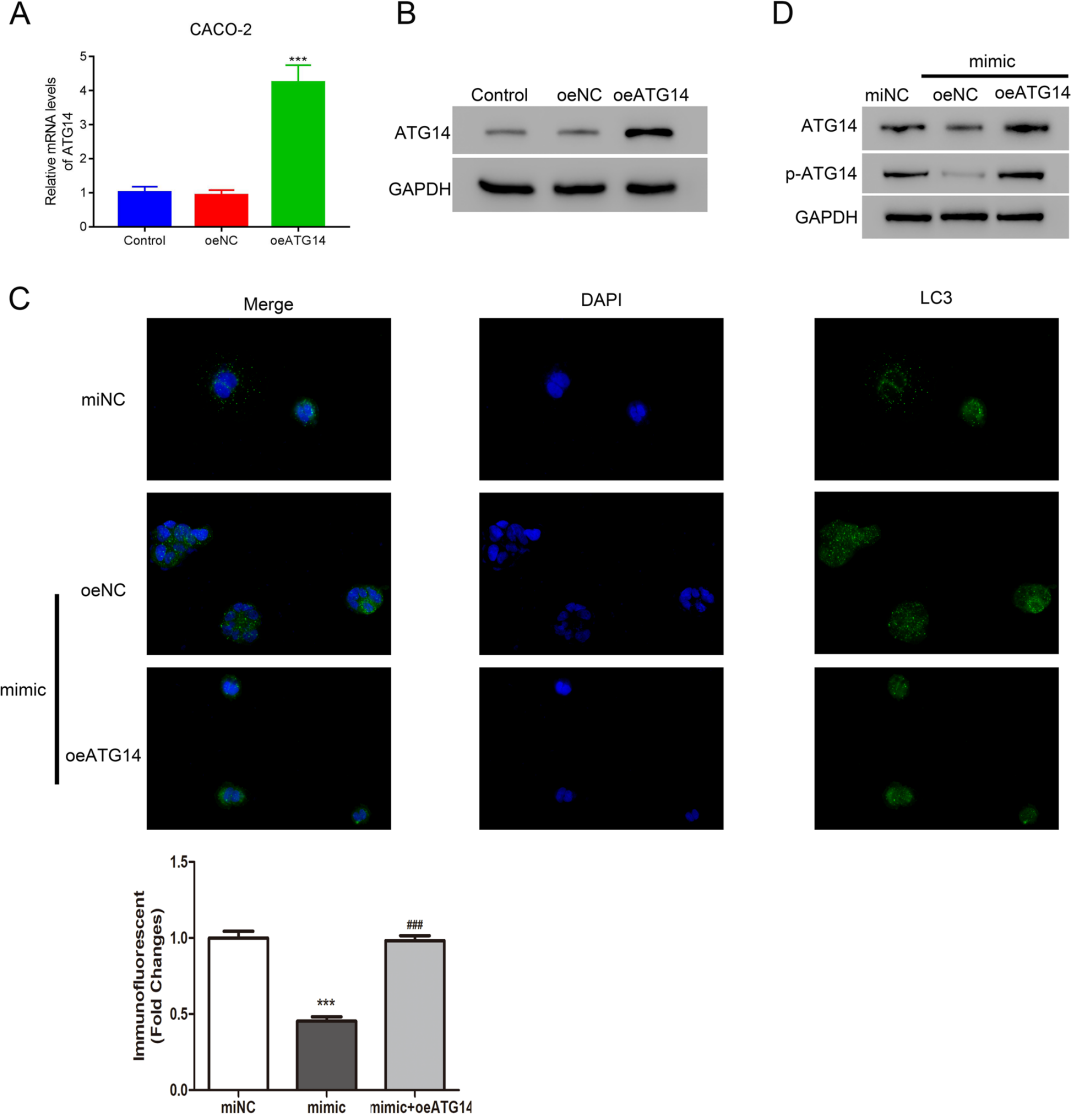
ATG14 overexpression promoted CACO-2 autophagy in the presence of miR-148b-3p mimic. **A**, **B** Relative mRNA and protein levels of ATG14 in CACO-2 cells after overexpressing ATG14. ***p < 0.001 vs oeNC. **C** Overexpression of ATG14 abolished miR-148b-3p mimic caused autophagy of CACO-2 cells. ***p < 0.001 vs miNC; ###*p* < 0.001 vs mimic. **D** Protein levels of ATG14 in CACO-2 cells after transfecting with oeNC or oeATG14 in the presence of miR-148b-3p mimic

To study the role of ATG14/miR-148b-3p in autophagy, ATG14 was successfully overexpressed in CACO-2 cells (Fig. 5A, B). IMF staining results indicated that overexpression of ATG14 abolished miR-148b-3p mimic caused autophagy of CACO-2 cells (Fig. 5C). Then, we examined the relative protein levels of ATG14. Western blots showed that overexpression of ATG14 reversed miR-148b-3p mimic caused a decrease of ATG14 (Fig. 5D). These results indicated that ATG14 overexpression promoted CACO-2 autophagy in the presence of miR-148b-3p mimic.

### ATG14 overexpression promoted the autophagy of CACO-2 cells in the presence of miR-148b-3p mimic through increasing ATG14

**Fig. 6:**
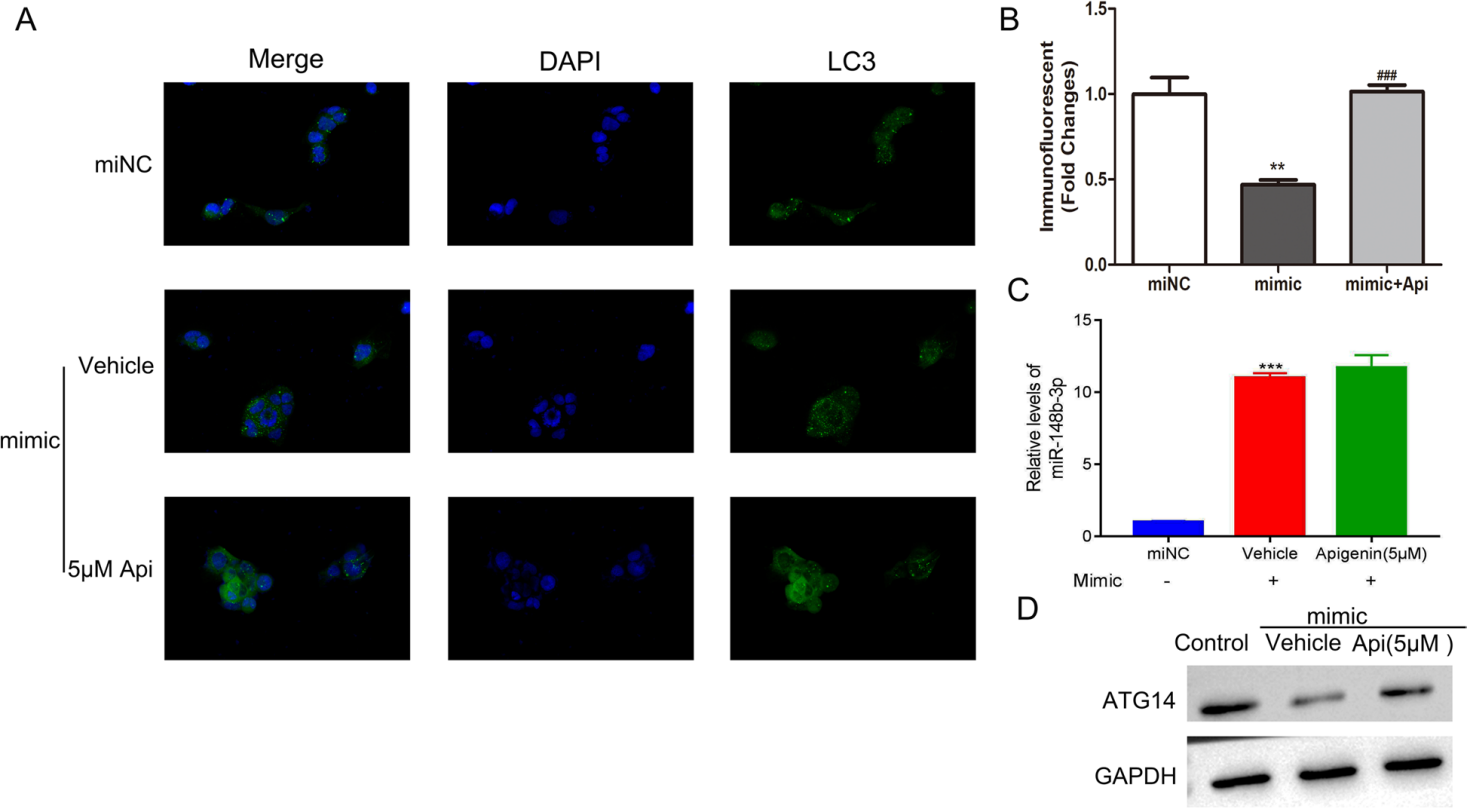
Overexpressing ATG14 promoted CACO-2 autophagy in the presence of miR-148b-3p mimic through increasing ATG14. **A**, **B** Apigenin promoted the autophagy of CACO-2 after co-cultured with miR-148-3p mimic. **p < 0.01 vs miNC; ###*p* < 0.001 vs mimic. **C** Apigenin did not affect miR-148b-3p in CACO-2 cells after co-cultured with miR-148b-3p mimic. **D** Apigenin treatment enhanced the protein level of ATG14 in CACO-2 cells in the presence of miR-148-3p mimic

To study the underlying mechanism by which ATG14 overexpression promoted CACO-2 autophagy, CACO-2 cells were treated by miR-148-3p mimic in the presence of apigenin. Results showed that apigenin promoted the autophagy of CACO-2 after co-cultured with miR-148-3p mimic (Fig. 6A, B). Q-PCR results indicated that apigenin did not affect miR-148b-3p in CACO-2 cells after co-cultured with miR-148b-3p mimic (Fig. 6C). However, apigenin treatment enhanced the protein level of ATG14 in CACO-2 cells in the presence of miR-148-3p mimic (Fig. 6D). Together, the data suggested that ATG14 overexpression promoted CACO-2 autophagy in the presence of miR-148b-3p mimic.

## Discussion

In this study, IBS-exos were successfully isolated and used to treat CACO-2 cells. We demonstrated that IBS-exos decreased the autophagy in CACO-2 cells. Administration of apigenin dose-dependently abolished the inhibitory effect of IBS-exo on CACO-2 autophagy. A mechanistic study indicated that miR-148b-3p suppressed ATG14 to suppress autophagy through the binding to its 3′UTR. In contrast, ATG14 overexpression promoted the autophagy of CACO-2 cells in the presence of miR-148b-3p mimic. These results identified a novel role of miR-148b-3p/ATG14 in CACO-2 autophagy and may facilitate the development of new drugs for IBS.

Apigenin plays a role in various diseases. For example, Malik et al. have demonstrated that apigenin ameliorated STZ-induced diabetic nephropathy [32]. Anusha et al. have reported that apigenin has a protective Parkinson’s disease via suppression of ROS-mediated apoptosis [33]. Apigenin has also been shown to suppress lupus [34]. The results indicate a key role of apigenin in the regulation of CACO-2 cell autophagy, showing for the first time that apigenin dose-dependently abolished the inhibitory effect of IBS-exos on the autophagy of CACO-2 cells.

miRNAs are small, noncoding RNA (21–25 nucleotides) that regulate gene expression [35]. Studies have shown that miRNA dysregulation regulates different biological processes [36, 37]. Kim et al. indicated that miR-148b-3p regulates angiogenesis and is a therapeutic candidate for overcoming endothelial cell dysfunction and angiogenic disorders [38]. Arambula-Meraz et al. have observed a correlation between miR-148b-3p with two established biomarkers of prostate cancer, PSA and PCA3, suggesting its potential as a biomarker of prostate cancer [39]. MiR-148b-3p has also been shown to inhibit the pro-angiogenic phenotype of endothelial cells [40]. This study further explored its biological function. We showed miR-148b-3p suppressed the transcription of ATG14 to suppress autophagy of CACO-2 cells through binding to its 3′UTR. These findings indicated a new function of miR-148b-3p in IBS, showing miR-148b-3p inhibited the autophagy of CACO-2 cells by suppressing ATG14 transcription through binding to its 3′UTR.

Our results indicated that miR-148b-3p bound directly to the 3′UTR of ATG14 promoter to negatively regulated ATG14 expression, which was verified by the facts that overexpressing ATG14 abolished miR-148b-3p-caused suppression of CACO-2 autophagy. The findings demonstrate for the first time that miR-148b-3p targets ATG14, and highlight the importance of miR-148b-3p/ATG14 signaling axis in human colon epithelial cells and IBS. Future studies in animals will provide more relevant data. Although shortcomings exist, this study demonstrated a new role of apigenin in the autophagy of CACO-2 cells.

## Conclusion

This study demonstrated a new function of apigenin in the autophagy of human colon epithelial cells, showing that apigenin dose-dependently abolished the inhibitory effects of IBS-exo on CACO-2 autophagy by regulating miR-148b-3p/ATG14 signaling.

## Data Availability

The datasets used and/or analyzed during the current study are available from the corresponding author on reasonable request.
